# Competitiveness and survival of two strains of *Glossina palpalis gambiensis* in an urban area of Senegal

**DOI:** 10.1371/journal.pntd.0006172

**Published:** 2017-12-27

**Authors:** Mireille Djimangali Bassène, Momar Talla Seck, Soumaïla Pagabeleguem, Assane Gueye Fall, Baba Sall, Marc J. B. Vreysen, Geoffrey Gimonneau, Jérémy Bouyer

**Affiliations:** 1 Institut Sénégalais de Recherches Agricoles, Laboratoire National d'Elevage et de Recherches Vétérinaires, Dakar–Hann, Sénégal; 2 Pan-African Tsetse and Trypanosomosis Eradication Campaign (PATTEC), Bobo-Dioulasso, Burkina Faso; 3 Ministère de l'Elevage et des Productions animales, Direction des Services Vétérinaires, Dakar, Sénégal; 4 Insect Pest Control Laboratory, Joint FAO/IAEA Programme of Nuclear Techniques in Food and Agriculture, Vienna, Austria; 5 Unité Mixte de Recherche ‘Interactions hôtes-vecteurs-parasites-environnement dans les maladies tropicales négligées dues aux trypanosomatides’, Centre de Coopération Internationale en Recherche Agronomique pour le Développement (CIRAD), Montpellier, France; 6 Unité Mixte de Recherche ‘Animal, Santé, Territoires, Risques et Ecosystèmes’, Centre de Coopération Internationale en Recherche Agronomique pour le Développement (CIRAD), Montpellier, France; University of Perugia, ITALY

## Abstract

**Background:**

In the Niayes area, located in the west of Senegal, only one tsetse species, *Glossina palpalis gambiensis* Vanderplank (Diptera: Glossinidae) was present. The Government of Senegal initiated and implemented an elimination programme in this area that included a sterile insect technique (SIT) component. The *G*. *p*. *gambiensis* strain (BKF) mass-reared at the Centre International de Recherche-Développement sur l'Elevage en zone Subhumide (CIRDES) in Burkina Faso was used for the SIT component.

**Methodology/principal findings:**

Studies conducted in 2011 in four localities in the Niayes area (Pout, Sébikotane, Diacksao Peul and the Parc de Hann) showed that the BKF strain demonstrated inferior survival in the ecosystem of the Parc de Hann, a forested area in the city centre of the capital Dakar. Therefore, *G*. *p*. *gambiensis* flies from the Niayes area (SEN strain) were colonized. Here we compared the competitiveness and survival of the two strains (BKF and SEN) in the Parc de Hann. Released sterile males of the SEN colony showed a daily mortality rate of 0.08 (SD 0.08) as compared with 0.14 (SD 0.08) for the BKF flies but the difference was not significant (p-value = 0.14). However, the competitiveness of the SEN males was lower (0.14 (SD 0.10)) as compared with that of the BKF males (0.76 (SD 0.11)) (p-value < 10^−3^).

**Conclusions/significance:**

Based on the results of this study, it can be concluded that the BKF strain will remain the main strain to be used in the elimination programme. Despite the slightly longer survival of the SEN males in the Parc de Hann, the superior competitiveness of the BKF males is deemed more important for the SIT component, as their shorter survival rates can be easily compensated for by more frequent fly releases.

## Author summary

Senegal is one of the many African countries infested by tsetse flies responsible for the transmission of trypanosomes to humans and animals, causing health and economic losses. In the Niayes area, located in the west of Senegal, only one tsetse species, *Glossina palpalis gambiensis* Vanderplank (Diptera: Glossinidae) is present, which is targeted by an elimination effort including the sterile insect technique (SIT). The quality of the strain used to implement any SIT effort is instrumental for its success. Here we compared the competitiveness and survival of irradiated males of two strains (BKF and SEN) released in the Parc de Hann, to assess their usefulness in eradicating this isolated population, located within the capital city, Dakar. We observed that the mating competitiveness of the SEN males was significantly lower as compared with that of the BKF males. Based on this study, we concluded that the BKF strain will remain the main strain to be used in the elimination programme in the Niayes.

## Introduction

Tsetse flies are the cyclical vectors of several trypanosomes, which cause human African trypanosomosis (HAT or sleeping sickness) and African animal trypanosomosis (AAT or nagana) [1]. AAT constitutes a major constraint to livestock development in sub-Saharan Africa [1], and the annual losses to African livestock caused by the disease is estimated at USD 4,750 million [2].

The Niayes area of Senegal, north east of Dakar, was infested by one tsetse species, *Glossina palpalis gambiensis* Vanderplank (Diptera: Glossinidae) [3]. In the 1970s a vector control programme was implemented in the Niayes, successfully suppressing the tsetse population [4]. However, this programme did not rely on area-wide principles and it failed to create a sustainable tsetse-free zone, with the re-introduction of flies from bordering infested areas as a consequence [5,6]. In 2005 the Government of Senegal, receiving financial and technical support from the Food and Agriculture Organization of the United Nations (FAO), the International Atomic Energy Agency (IAEA), the Centre de Coopération Internationale en Recherche Agronomique pour le Développement (CIRAD), and the Department of State of the United States under the Peaceful Uses Initiative (PUI), embarked on a tsetse eradication programme under the umbrella of the Pan African Tsetse and Trypanosomosis Eradication Campaign (PATTEC). The programme was implemented following area-wide integrated pest management (AW-IPM) principles that combined the use of insecticide-impregnated traps and targets and insecticide pour-on on livestock with the release of sterile male insects (Sterile Insect Technique (SIT)) (http://projet-tsetse-niayes.cirad.fr/).

During the first phase of the project, several baseline data sets were collected as part of a feasibility study which indicated that *G*. *p*. *gambiensis* was the only tsetse species present in the target area [6], and that the tsetse population of the Niayes was genetically isolated from the main tsetse belt located more than 200 km to the south in Senegal [7]. Moreover, it was proposed to procure the sterile flies from the Centre International de Recherche-Développement sur l'Elevage en zone Subhumide (CIRDES), Bobo Dioulasso, Burkina Faso, where a colony of the same species has been maintained for more than 35 years. Studies conducted in walk-in field cages indicated that there were no mating barriers between the mass-reared strain from Burkina Faso (BKF) and the strain from the Niayes (SEN) [8]. In addition, the local tsetse populations seem to have adapted to a peri-urban ecosystem, i.e. the Parc de Hann which is located within the densely populated city of Dakar.

The decision was taken to develop a local SEN strain to serve as a back-up in case the BKF strain would not perform in certain ecosystems of the target area. Pupae derived from females collected in Pout were used to initiate a SEN colony at the Insect Pest Control Laboratory (IPCL) of the Joint FAO/IAEA Programme of Nuclear Techniques in Food and Agriculture, Seibersdorf, Austria that was later transferred to the Slovak Academy of Sciences (SAS), Bratislava, Slovakia for amplification.

During pilot release trials in various ecosystems in the Niayes, survival of the sterile males of the BKF strain was inferior in the Parc de Hann as compared with their survival in the other ecosystems [9]. Therefore, this study was undertaken to compare the competitiveness and survival of the SEN and BKF strain in the Parc de Hann, this being one of the most anthropized sites of the target area. The results of the study would provide guidance to decide which of the two strains would be indicated for use in the elimination campaign in this special ecosystem.

## Materials and methods

### Study area

**Fig 1:**
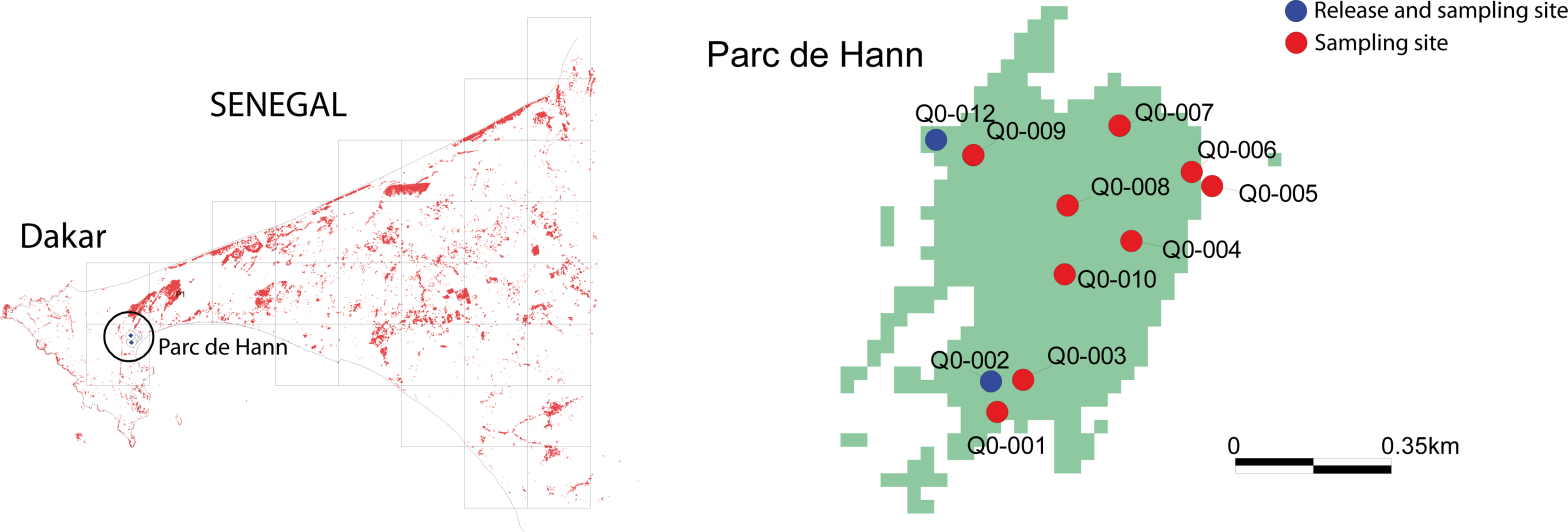
Study area indicating the release and sampling sites. On the left panel, grid cells represent 5*5 km squares in the Cap Vert peninsula around Dakar, Senegal. The Parc de Hann is indicated by the black circle.

The study was conducted in the Parc de Hann (17° 43 W and 14° 72 N), an area of 60 ha in the city centre of Dakar (Fig 1) with a habitat consisting of wooden savannah, woodland and a permanent lake surrounded by reeds and a swampy forest. Animals such as lions, chimpanzees, jackals, buffalos and numerous birds are present in the Zoological Park and serve as a host for the local tsetse population. The park is highly frequented by people; to protect visitors, it is often subject to insecticide treatment and bush clearing in order to control biting insects. Plant nurseries are present with various domestic and exotic species.

### Origin of the two tsetse strains

The BKF strain and the SEN strain were the two strains of *G*. *p*. *gambiensis* used in this study. The BKF strain was developed from pupae collected in Guinguette, near Bobo-Dioulasso, Burkina Faso in 1972, and since then the colony has been maintained at the CIRDES. In 2009, pupae were shipped from the CIRDES to the IPCL where a second colony of this strain was developed, and later a third colony in the SAS. The SEN strain originated from flies caught in the area of Pout, Senegal in 2010 and was initiated at the IPCL and amplified at the SAS [10].

In both the CIRDES and the IPCL (SAS pupae were irradiated in the IPCL), four-week old pupae (29 to 30 days old) of *G*. *p*. *gambiensis* were irradiated with a dose of 110 Gy under chilled (4–6°C) conditions to lower their metabolic rate, preventing emergence. This was done just after the first males emerged, taking profit of protogyny (females emerge first) in tsetse to sort sexes and resulting in less than 2% of females in the chilled pupae. Irradiated pupae were then packed in insulated isotherm boxes before shipment by air to the Laboratoire National de l’Elevage et de Recherches Vétérinaires (LNERV) in Dakar [11]. The transport duration was on average 36h and 48h for the shipments originating from CIRDES and SAS, respectively.

Upon arrival at the LNERV, the pupae were transferred to the insectary, put in Petri dishes and covered with sand mixed with fluorescent dye powder (0.25%; DayGlo) to mimic natural emergence conditions in the soil. During emergence, the fluorescent dye was sequestered in the ptilinum so that flies trapped in monitoring traps could be examined under a UV microscope to allow discrimination of wild from sterile flies [12]. Different fluorescent dyes (red, yellow, blue, orange) were used for different release weeks.

From each batch of received pupae (SEN and BKF strain), a sample of 50 pupae was taken for a standardized quality control test. The quality control pupae were kept under the same environmental conditions as the original batch of pupae, but the Petri dish with the pupae was put in a flight cylinder, i.e. a PVC tube 10 cm high and 8.4 cm in diameter. The inner wall of the cylinder was coated with unscented talcum powder to prevent the flies from crawling out [11]. This protocol allowed an assessment of the emergence rate and the propensity of the sterile male flies to fly out of the cylinder. Only those flies that managed to escape the flight cylinder after emergence were considered as “operational flies” [11].

Emerged flies were sorted and classed as “normal” (i.e. flies with no apparent morphological deficiencies, even if some of them had no ability to fly) or “abnormal flies” (i.e. with deformed wings). Non-emerged pupae were also identified and counted. The emerged normal flies were given a blood meal for 3–5 days before release, using a silicone membrane “*in vitro*” feeding system [13]. The bovine blood meal was mixed with the trypanocidal drug Trypamidium (Samorin) at a dose of 10mg/l [14,15] to avoid cyclical development of trypanosomes in the released sterile males. Fly emergence rate and proportion of normal males were calculated before release in order to determine the quality and quantity of the flies destined to be released.

### Baseline entomological survey: Tsetse density and abortion rate

Entomological surveys were conducted in the park to assess natural abortion rates in May 2009 and 2015. Ten biconical traps [16] interspaced by 100 to 300 meters were deployed for 72 hours, with flies sampled every 24 hours. The traps received an identification code and were geo-referenced (Fig 1). Trapped flies were identified and counted by species and sex, and apparent density (number of flies per trap per day (ADP)) was calculated [17]. All trapped live and freshly dead female flies were dissected to determine their physiological age and reproductive status (gravid or not) [18,19]. The rate of induced sterility was determined, taking into consideration the status of the uterus and the size of the next follicle in ovulation sequence. Females with either a degenerated egg *in utero*, or an empty uterus with an immature follicle next in ovulation sequence, were considered as having mated with a sterile male [18,20].

### Release of sterile males

Four to 5 day old sterile male flies were released every week on Friday afternoon (around 05:00 PM) from June to September 2011 for the BKF strain and from June to October 2015 for the SEN strain. Flies were transported to the release sites in Roubaud cages (4.5 × 13 × 8 cm) [21] that were covered with tulle (1 mm × 1 mm mesh), each containing at least 50 sterile male flies. Cages were kept in climate controlled containers (temperature 24–26°C and humidity 75 ± 5%) during the transport to the release sites.

Flies were released in two release sites, one located in favourable habitat and the other in a site with unfavourable vegetation cover [6]. A dense *Euphorbia* hedge with natural woodland that had a tree cover between 50 and 75% was present in the favourable site, whereas the trees in the unfavourable site had less than 50% tree cover and contained *Euphorbia* and cashew nuts [6]. For each release, flies were dyed with different colours corresponding to the two release sites. At the end of the release, the number of non-flying flies was recorded. Note that the number of released flies varied among release session according to the number of pupae received and their quality (i.e. rate of operational flies).

### Competitiveness and survival of sterile males

The dynamics of the sterile to wild male ratio and induced sterility in native female flies were monitored through regular trapping sessions on day 3, 5 and 7 after each sterile male release. Ten biconical traps were deployed in the same 10 geo-referenced sites as used during the entomological baseline data collection (Fig 1). Traps were deployed in the morning at 8am and checked at 12am and 3pm the same day; trapped tsetse flies were counted, sexed according to genitalia differences and dissected. The heads of all caught male flies were removed from the thorax, glued on paper and observed under an UV camera to discriminate sterile (stained with fluorescent dye) from wild males (not stained), enabling the calculation of the sterile to wild male ratio [12]. All trapped live female flies were dissected and their reproductive status assessed [18] to calculate the abortion rate of females.

### Statistical analyses

The competitiveness of sterile males was determined using the Fried index [22]. This index compares abortion rates in wild females before and during the release of sterile males, taking into account the sterile to wild male ratio. This competitiveness is calculated using the following formula: $c=\frac{(Ha-E)/E}{S/N}$ modified from the Fried competitiveness index, with Ha being the percentage of fertile females prior releases, E the percentage of fertile females during the release period for the same seasonal period, and S/N the sterile to wild male ratio. The Hs factor present in the original formula [22] that denotes hatch rate from eggs of females mated with sterile males was omitted because in this species, males irradiated with more than 100 Gy at the pupal stage can be considered as fully sterile [23].

The daily mortality rate (μ) was calculated after each release session. Thus, for a given session, the number of trapped sterile flies (N) was recorded by date of trapping. The daily survival rate of each series was estimated as the exponential of the slope of the curve of ln (N) ~ time and μ = 1- (survival rate). The average lifespan or half-life of the flies was deduced from the daily mortality and corresponded to -1n(2) / ln(1-μ).

The natural abortion rates between the two study periods was subjected to a Chi-square test comparison.

A generalized linear model using month and strain as explanatory variables was used to compare the competitiveness and mortality rates of the BKF and SEN strain. The R Software (version 3.1.0) package was used to carry out all statistical analyses [24].

### Ethical statement

The study was conducted in the framework of the tsetse elimination campaign in Senegal, implemented by the Direction des Services Vétérinaires, Ministère de l’Elevage and the ISRA (Institut Sénégalais de Recherches Agricoles)/LNERV (Laboratoire National d’Elevage et de Recherches Vétérinaires), Ministère de l’Agriculture et de l’Equipement Rural. This project received official approval from the Ministère de l’Environnement of Senegal, under the permit N°0874/MEPN/DE/DEIE/mbf.

## Results

### Natural abortion rate and density before release

In May 2009, 24 females were dissected of which 4 showed signs of an abortion, whereas in May 2015, 32 females were dissected of which only 2 showed signs of an abortion. The two abortion rates were not significantly different (χ^2^ = 0.66, df = 1, p-value = 0.42) and we thus considered 10.7% (SD 4.1%) as the baseline natural abortion rate.

The apparent densities of wild flies the month before releases were also very similar between the two study periods, with 1.49 (SD 1.15) and 1.91 (SD 1.68) flies per trap per day in May 2011 and 2015 respectively.

### Quality of released sterile males

From May to September 2011, 94,961 irradiated pupae of the BKF strain were received at the LNERV (74,495 pupae from the CIRDES and 20,444 pupae from the SAS), of which on average 66.0 ± 15.0% and 67.5 ± 12.3% emerged respectively. The proportion of flies with undeployed wings was different depending on the origin (p-value <10–3) with 11.0 ± 4.5% and 20.2 ± 7.4% for the CIRDES and SAS flies, respectively. The rate of operational males (i.e. normal flies with ability to fly) was also different between origin (p-value = 0.04) with an average of 53.9 ± 14.1% for the CIRDES flies and 43.6 ± 13.7% for the SAS flies. A total of 12,191 sterile male flies of the BKF strain were released (excluding non-flyers) from the ground in the Parc de Hann between May and October 2011. After the release, on average 7.9 ± 3.9% of live flies remained in the release boxes, although their wings were visually normal.

From May to September 2015, a total of 12,032 irradiated pupae of the SEN strain were received at the LNERV. All the chilled irradiated pupae originated from the insectary in the SAS. On average 60.7 ± 28.0% of the flies emerged of which 55.5% were sterile males. The rate of operational sterile male flies was 86.1% and a total of 4264 flies of the SEN strain were released (excluding non-flyers) in the Parc de Hann in 2015, of which 13.84% were non fliers.

### Competitiveness of sterile BKF and SEN males

A total of 440 and 354 wild females were caught in the Parc de Hann from June to September 2011 and 2015 respectively (Table 1).

**Table 1:** Competitiveness of two strains of *Glossina palpalis gambiensis* in the Parc de Hann, Senegal.

**SEN strain**								
**Month**	**Nb** **aborted**	**Nb** **dissected**	**Pcent** **aborted**	**Nb** **released**	**Wild (W)** **males**	**Sterile (S)** **males**	**Ratio S/W**	**Compet**
June	10	78	12.82	319	49	36	0.734	0.033
July	13	81	16.05	1777	55	27	0.490	0.130
August	15	105	14.28	1247	74	26	0.351	0.119
September	14	90	15.55	636	68	14	0.205	0.280
**BKF strain**								
**Month**	**Nb** **aborted**	**Nb** **dissected**	**Pcent** **aborted**	**Nb** **released**	**Wild** **males**	**Sterile** **males**	**Ratio S/W**	**Compet**
June	21	47	44.68	1575	64	56	0.875	0.702
July	44	132	33.33	2085	48	25	0.520	0.653
August	80	211	37.91	6927	123	70	0.569	0.770
September	26	50	52.00	1604	68	65	0.955	0.901

Monthly monitoring results and the calculated competitiveness are presented for the period June to September 2015 for the SEN strain and from June to September 2011 for the BKF strain (Nb = Number; Pcent = percentage; Compet = Competitiveness).

Trapped female flies were dissected and the data indicated that the BKF sterile males induced an average monthly sterility of 42.0% in the native wild female population, with a maximum of 52.0% in September and a minimum of 33.3% in July (Table 1). The trapping data indicated a mean sterile to wild male ratio of 0.73:1 with a maximum of 0.95:1 observed in September and a minimum of 0.52:1 in July. The competitiveness of the BKF strain, as indicated by the Fried index, was on average 0.76 (SD 0.11).

The average induced sterility of the wild female population during the release period of the SEN males was 14.7% with a maximum of 16.0% observed in July and a minimum of 12.8% in October (Table 1). The trapping data indicated a mean sterile to wild male ratio of 0.45:1 with a maximum of 0.73:1 observed in June and a minimum of 0.20:1 in October. The average Fried competitiveness index of the SEN strain was 0.14 (SD 0.10).

**Fig 2:**
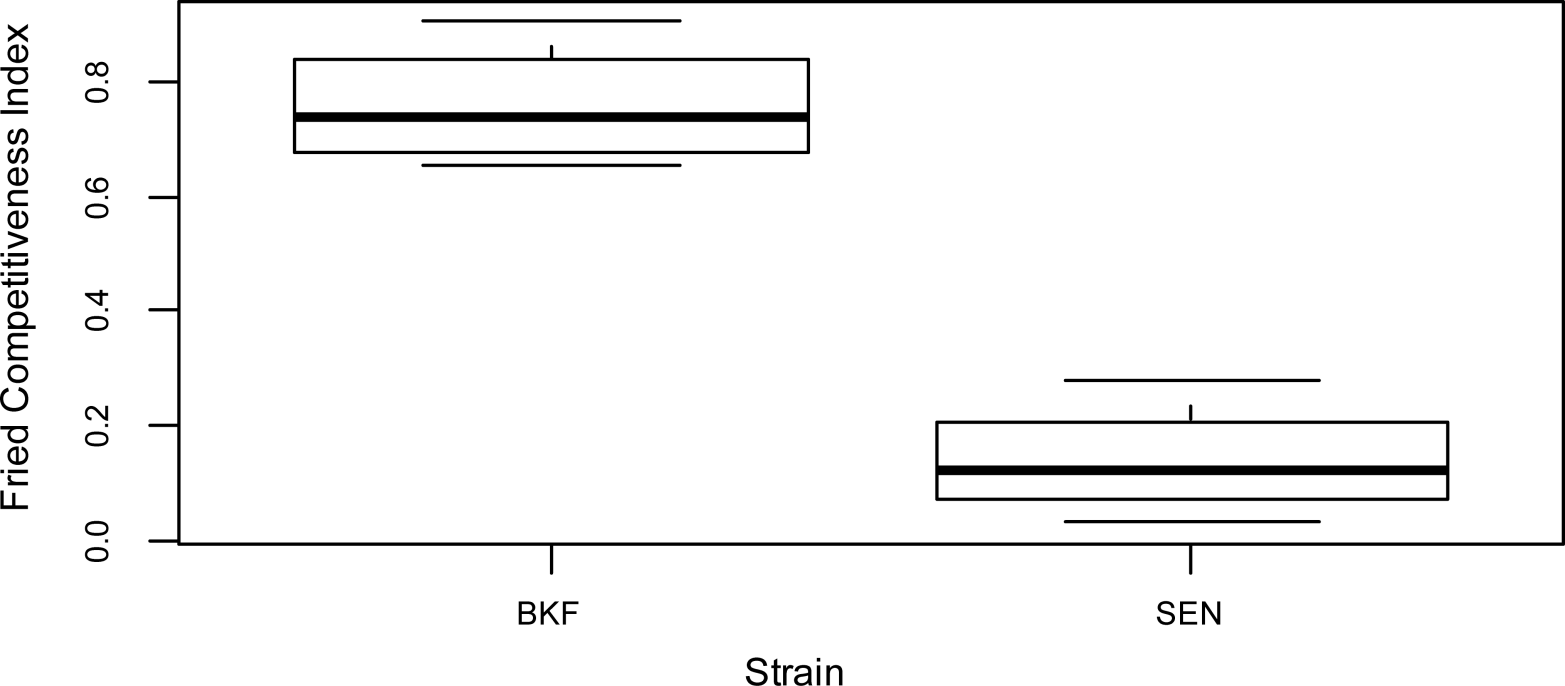
Average Fried index per month of two strains of *Glossina palpalis gambiensis* in Senegal. Sterile BKF and SEN males were released in the Parc de Hann of Dakar from June to September 2011 and 2015 respectively.

The BKF sterile males were significantly more competitive than the sterile males of the SEN strain (Fig 2, p-value < 10^−3^). The competitiveness of sterile males was higher in September than for the other months for both strains (p-value = 0.017).

### Survival of the BKF and SEN strain

**Fig 3:**
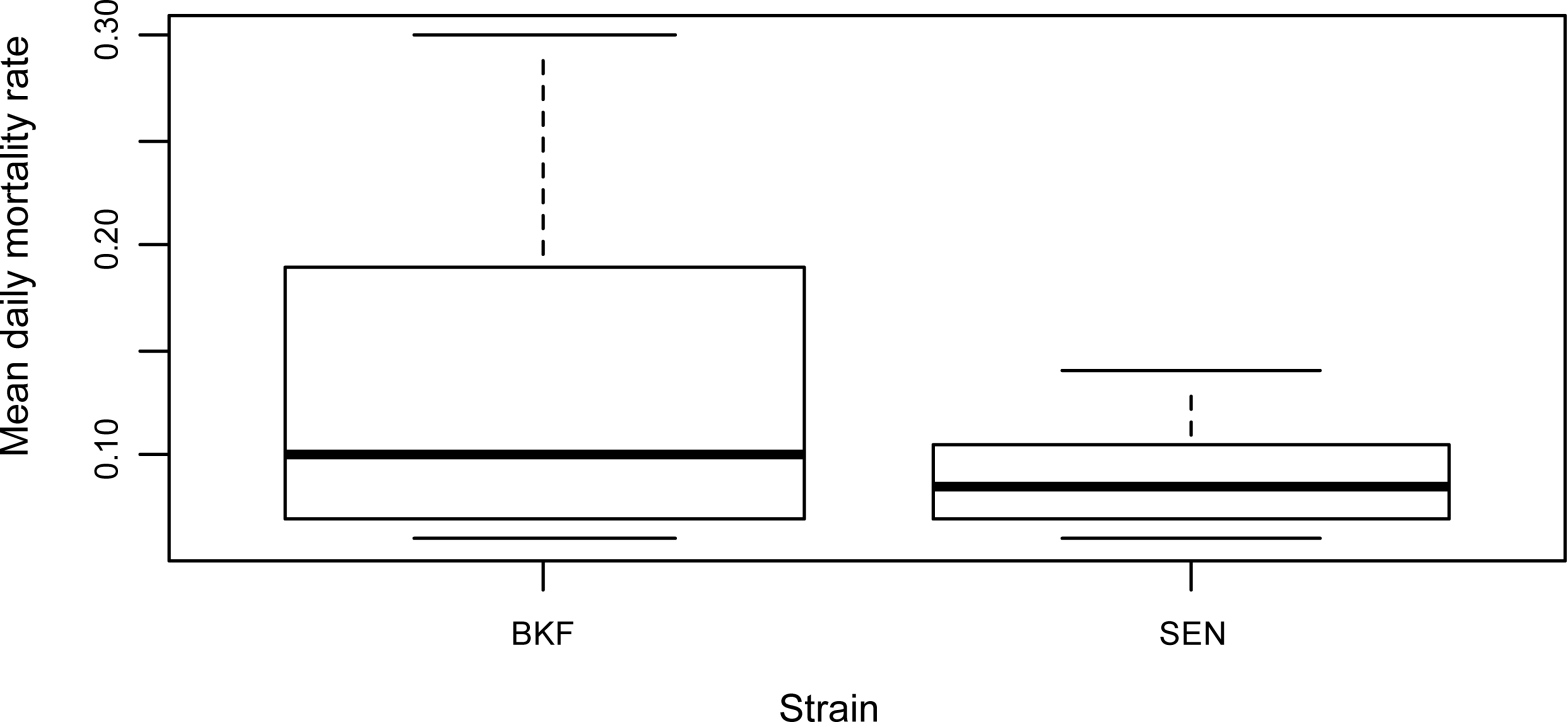
Mean daily mortality rate of two strains of *Glossina palpalis gambiensis* in Senegal. BKF and SEN sterile males were released in the Parc de Hann from June to October 2011 and June to September 2015 respectively.

Table 2 presents the daily mortality rates of the sterile males of the two strains for those release sessions where an estimation of the mortality was possible. The mean lifespan of the BKF males was 9.7 days (SD 5.0) whereas it was 11.9 days (SD 2.9) for the SEN males. The average daily mortality rate was 0.08±0.08 for SEN males and 0.14±0.08 for BKF males, which was not significantly higher (p-value = 0.14) (Fig 3). The differences between months were not significant (p-value > 0.21).

**Table 2:** Daily mortality of two strains of *Glossina palpalis gambiensis* in the Parc de Hann, Senegal. Two batches of flies with different colours were released when the number of pupae was large enough, in order to make two measures of the mortality rate.

Date	nb released	Mortality rate	Strain
12/06/15	319	0.07	SEN
19/06/15	209	0.1	SEN
10/07/15	326	0.08	SEN
10/07/15	351	0.11	SEN
17/07/15	121	0.14	SEN
24/07/15	238	0.06	SEN
31/07/15	86	0.09	SEN
21/08/15	209	0.12	SEN
21/08/15	230	0.07	SEN
28/08/15	225	0.07	SEN
18/09/15	174	0.09	SEN
18/09/15	184	0.07	SEN
27/05/11	1432	0.09	BKF
27/05/11	1639	0.24	BKF
24/06/11	1575	0.06	BKF
29/07/11	2085	0.09	BKF
12/08/11	1790	0.11	BKF
12/08/11	1614	0.07	BKF
26/08/11	1896	0.19	BKF
26/08/11	1627	0.3	BKF
09/09/11	1604	0.16	BKF

Daily mortality rates of BKF and SEN strains were measured from May to September 2011 and June to October 2015 respectively.

## Discussion

The biological quality of the sterile male insects that are being released over the target area is a key component for the success of an operational AW-IPM programme that includes a SIT component [25]. The released insects must have essential biological characteristics such as mobility, dispersal, survival, mating compatibility and competitiveness that should be as close as possible to that of the wild insects [26,27]. The two *G*. *p*. *gambiensis* strains used in the study, i.e. one originating from Burkina Faso (BKF) and the second one originating from Senegal (SEN), had already been tested for mating compatibility in walk-in field cages and no barriers to mating were detected with local flies from the release area [8]. This mating study indicated that it might be feasible to use sterile males from the BKF strain for release in the Niayes area of Senegal. A BKF colony of significant size was maintained at the CIRDES in Burkina Faso.

However, during pilot release trials with BKF males in various ecosystems in the Niayes, it was noticed that their survival in the Parc de Hann was inferior to their survival in other ecosystems of the Niayes [9]. Therefore, it was deemed necessary to assess whether the males derived from the SEN strain would perform better in the Parc de Hann. This study was therefore designed to assess the competitiveness and survival of the males of the two strains in the Parc de Hann. The data indicate that the SEN males survived on average longer in comparison with the BKF males, albeit non significantly, whereas the BKF males were on average much more competitive for mating with wild females as compared to the SEN males.

The BKF and SEN flies were produced in different mass-rearing facilities but showed similar emergence rates from pupae upon arrival at the LNERV, i.e. 60.7 ± 28.0% and 66 ± 15.0% respectively. Although rearing procedures and transport conditions were similar between the two strains [28,29], important differences in quality were observed. The percentage of flies with undeployed wings was significantly lower for the BKF flies (11.0 ± 4.5%) as compared to the SEN flies (20.2 ± 7.4%). In addition, the percentage of operational sterile males was significantly higher when they originated from BKF pupae, as compared to SEN pupae (53.9 ± 14.1% for CIRDES pupae and 43.6 ± 13.7% for SAS pupae), which seems to indicate a better inherent quality of the BKF flies. These differences may be attributed to potential factors experienced during the transport, such as mechanical shocks and the duration of the chilling, that might have a negative impact on the quality of the emerging flies, all the more than SEN males were transported 24H longer than BKF males [11]. The observed differences in competitiveness might be partially due to these differences in quality but also to differences in reproductive behaviour (i.e. aggressiveness, courtship…).

Entomological surveys conducted in the Parc de Hann in May 2009 and 2015 in the absence of any release of sterile males indicated that the natural abortion rate of the native *G*. *p*. *gambiensis* population in the Parc de Hann was not significantly different between both periods. This was an important result that allowed a comparison of the competitiveness of the sterile males from the two strains during two different periods. However, it must be acknowledged that differences in climatic conditions or other environmental parameters like insecticide spraying might have caused the observed differences, all the more as the natural abortion rate was estimated from a small amount of females before releases. Actually, the “natural” rate of abortion may vary over time as the embryo or developing larvae may be aborted at any time during development if the female is stressed (e.g. if food is in short supply, environmental conditions are not ideal or insecticides are used) [30]. The vegetation was however very similar between the two periods, since the study was conducted in a protected area, and the flies can move easily 50 to 100m to find their best resting sites in term of climatic conditions (ecidioclimate) [30]. Finally, the apparent density of the wild population and the ratio of sterile to wild males were similar between the two trials and cannot explain the observed differences.

The dissection data indicated that the males of the BKF strain were significantly more competitive than the native SEN strain with a mean Fried index of 0.85 (SD 0.40) for the BKF males and 0.31 (SD 0.25) for the SEN males. This indicates that, although the BKF strain has been cultured for more than 35 years, the sterile males were still competitive in the ecosystem of the Parc de Hann. This strain was even more competitive in this study as compared to what was measured recently in Burkina Faso, when a much lower Fried index (0.07 (SD 0.02)) of the BKF males was obtained when released in a gallery forest inhabited by a local *G*. *p*. *gambiensis* population. Although the BKF and SEN strains are not incompatible [31], laboratory data from cross-mating experiments revealed a significant reduction of the fecundity of the hybrid offspring [32], which did not correlate with a decreased competitiveness of BKF males toward the local population. Although the long rearing history of several insects generally leads to a loss of competitiveness, the reverse effect was observed in this study. We do not have a good explanation for this result, which might be due to intrinsic differences in mating aggressiveness of the two strains. Moreover, a comparative study of the life history traits of BKF and SEN in relation to temperature and hygrometry revealed significant differences, with BKF flies being more resilient to high temperatures [10]. The two strains have different ecological affinities related to different native habitats; the BKF flies living mainly in riparian forests in Burkina Faso, whereas rivers have disappeared in the Niayes area and the SEN flies are mainly associated with anthropic tree habitats and relics of riverine thickets (see below) [33,34]. The populations of the Niayes and those of Burkina Faso are separated by natural barriers preventing gene flow [7] which allowed them to evolve independently even if they still are considered as the same species [35]. Therefore, the SEN strain should be more competitive in its specific environment but the results observed, with the better competitiveness of the BKF strain, probably reflect intrinsic differences in reproductive behaviour, independently from the environment, or an environmental effect that is not understood at this stage. Males of the BKF strain released in the Parc de Hann still had on average a shorter lifespan than their counterparts from the SEN strain. This would seem logical as the males of the SEN strain were developed from a local population in Senegal, which was highly adapted to the specific environment of the Niayes. These local ecological conditions of the Niayes are completely different from the riparian forests in Burkina Faso that are the natural habitat of the BKF flies [36]. The *G*. *p*. *gambiensis* population of the Niayes thrived in man-made ecosystems where the vegetation is dominated by mango, citrus and palm tree plantations that are interspaced with sparse residual riparian thickets [6,37,38]. The Parc de Hann is even more exceptional in that it is a forested area with caged wild animals located in the city of Dakar with a strong anthropic pressure [6]. Its specific characteristics were most likely reflected in the slightly increased average lifespan of the SEN males. Factors other than temperature, e.g. air pollution, may be responsible for this result, as a recent study that assessed survival of the BKF and SEN strains under laboratory conditions showed that males of the BKF strain survived significantly longer than males of the SEN strain at high temperatures [10]. This result is most likely related to the long colonization time of the BKF strain (>35 years), resulting in flies have adapted very well to the specific environmental conditions of the insectary, with few variations of the abiotic holding conditions. Conversely, the SEN strain had only been cultured for four years at the time of the study and therefore is much less adapted to the artificial environment of the laboratory. The observed differences in strains survival (i.e. lower collection of males) might also result from a greater dispersal ability of the BKF strain. However, this hypothesis seems unlikely because the Parc de Hann is enclosed in a urban area that is unfavourable to tsetse flies, limiting their dispersal outside the park.

Based on the data of this study, it can be concluded that the BKF strain will remain the main strain used in the elimination programme in the Niayes. Despite the slightly longer survival of the SEN males in the Parc de Hann, the superior competitiveness of the BKF males (as an indication of its satiric behaviour) is deemed more important for the SIT component, as their shorter survival rates can be easily compensated for with more frequent release sessions. In addition, the BKF strain has a much better fecundity under artificial rearing conditions as compared with the SEN strain, which will provide more sterile males for the same rearing effort, making the SIT more cost-effective. The use of spatio-temporal population models that are under development within the project will provide additional guidance on the relative importance of survival versus competitiveness. This study demonstrates the importance to conduct baseline entomological studies prior to any SIT program [39,40] in order to fully understand the bio-ecology and behaviour of the species studied. Moreover, it shows the importance of testing the strain intended to be used for the operational phase of any eradication program, especially in specific ecosystems, since the results are hardly predictable from laboratory data. The availability of the best strain to be used and its mass rearing capacity is also important to assess before starting the operational phase.
